# Role of key-regulator genes in melanoma susceptibility and pathogenesis among patients from South Italy

**DOI:** 10.1186/1471-2407-9-352

**Published:** 2009-10-03

**Authors:** Milena Casula, Antonio Muggiano, Antonio Cossu, Mario Budroni, Corrado Caracò, Paolo A Ascierto, Elena Pagani, Ignazio Stanganelli, Sergio Canzanella, MariaCristina Sini, Grazia Palomba, The Italian Melanoma Intergroup (IMI), Giuseppe Palmieri

**Affiliations:** 1 Istituto di Chimica Biomolecolare, Consiglio Nazionale delle Ricerche, Sassari, Italy; 2 Oncologia Medica 1, Ospedale Oncologico Businco, Cagliari, Italy; 3 Servizio di Anatomia Patologica, Azienda Ospedaliero Universitaria, Sassari, Italy; 4 Servizio di Epidemiologia, Azienda Sanitaria Locale 1, Sassari, Italy; 5 Istituto Nazionale Tumori Fondazione Pascale, Napoli, Italy; 6 Istituto Dermopatico dell'Immacolata, Roma, Italy; 7 Istituto Tumori Romagna, Meldola, Forli, Italy; 8 Associazione House Hospital Onlus, Napoli, Italy

## Abstract

**Background:**

Several genetic alterations have been demonstrated to contribute to the development and progression of melanoma. In this study, we further investigated the impact of key-regulator genes in susceptibility and pathogenesis of such a disease.

**Methods:**

A large series (N = 846) of sporadic and familial cases originating from South Italy was screened for germline mutations in *p16*^*CDKN*2*A*^, *BRCA2*, and *MC1R *genes by DHPLC analysis and automated DNA sequencing. Paired primary melanomas and lymph node metastases from same patients (N = 35) as well as melanoma cell lines (N = 18) were analyzed for somatic mutations in *NRAS*, *BRAF*, and *p16*^*CDKN*2*A *^genes.

**Results:**

For melanoma susceptibility, investigations at germline level indicated that *p16*^*CDKN*2*A *^was exclusively mutated in 16/545 (2.9%) non-Sardinian patients, whereas *BRCA2 *germline mutations were observed in 4/91 (4.4%) patients from North Sardinia only. Two *MC1R *germline variants, Arg151Cys and Asp294His, were significantly associated with melanoma in Sardinia. Regarding genetic events involved in melanoma pathogenesis at somatic level, mutually-exclusive mutations of *NRAS *and *BRAF *genes were observed at quite same rate (about two thirds) in cultured and *in vivo *melanomas (either primary or metastatic lesions). Conversely, *p16*^*CDKN*2*A *^gene alterations were observed at increased rates moving from primary to metastatic melanomas and melanoma cell lines. Activation of the ERK gene product was demonstrated to be consistently induced by a combination of molecular alterations (*NRAS*/*BRAF *mutations and *p16*^*CDKN*2*A *^silencing).

**Conclusion:**

Our findings further clarified that: *a*) mutation prevalence in melanoma susceptibility genes may vary within each specific geographical area; *b*) multiple molecular events are accumulating during melanomagenesis.

## Background

In fair skinned populations, both incidence and mortality rates of melanoma have been increasing over the past decades [1]. Fortunately, the most recent data on the melanoma epidemic suggest that majority of melanoma patients presents with thin lesions (Breslow thickness ≤ 1.00 mm) at the time of diagnosis, due to a steady improvement of the secondary prevention and early detection [2].

Baseline melanoma incidences have been found to vary according to the population's origin: Australia, the United States, and Sweden present a higher baseline incidence than Europe (except Sweden) [3,4]. Considering the European population, there is a gradient of melanoma incidence moving from northern countries (where incidence is higher) to southern countries [5]. In Italy, different incidence rates of melanoma between the northern and southern parts of the country (standardized rates per year per 100.000 inhabitants: 10.5-13.5 in North Italy versus 3.5-4.5 in South Italy) have been also reported [5].

Environmental and, mostly, genetic factors have been demonstrated to participate in susceptibility and pathogenesis of human melanoma. An intermittent exposure to ultraviolet radiation, especially in combination with endogenous factors like skin type and number of nevi, is the most commonly involved environmental factor [6-9].

From the genetic point of view, several gene alterations have been implicated. Germline mutations in *p16*^*CDKN*2*A *^gene represent the most recognized cause of inherited melanoma susceptibility. Prevalence of *p16*^*CDKN*2*A *^mutations seems to be heterogeneously distributed among melanoma patients within different geographical areas [10]; our previous study on melanoma patients originating from South Italy indicated that *p16*^*CDKN*2*A *^mutations were absent in North Sardinia and occurred in non-Sardinian patients only [11]. Several low-penetrance candidate genes, such as *breast cancer susceptibilitygene 2 *(*BRCA2*) and *melanocortin-1-receptor *(*MC1R*), have been also implicated in melanoma predisposition [12,13]. Inherited mutations of the *BRCA2 *gene have been associated to development of both ocular and cutaneous melanomas, in addition to the main predisposition to breast and ovarian cancers [12-14]. The *MC1R *gene encodes the melanocyte-stimulating hormone receptor (MSHR), a member of the G-protein-coupled receptor superfamily and represents one of the major genes which determine skin pigmentation [15-17]. The *MC1R *gene is remarkably polymorphic in Caucasian populations [18]; its sequence variants can result in partial (r) or complete (R) loss of the receptor's signalling ability [19]. The *MC1R *variants were suggested to be associated with red hair, fair skin, and increased risk of both melanoma and non-melanoma skin cancers [18-21]. Moreover, melanomas developed on skin not chronically exposed to sun usually carry either a mutated *NRAS *or mutated *BRAF *or concurrently mutated *BRAF *and *PTEN *[22]; evidence indeed suggest that *BRAF/PTEN *and *NRAS *somatic mutations are mutually exclusive or, in other words, that both mutation subsets does not occur in the same melanoma cell [22-25]. Finally, the ERK1-2 proteins, which represent the downstream components of the NRAS-BRAF-MEK signaling kinase cascade, have been found to be activated through phosphorilation (pERK_1-2_) in melanoma and implicated in rapid malignant cell growth, mostly as a consequence of mutations in upstream components of the pathway [25-27].

The aim of the present study was to evaluate the spectrum of such genetic alterations in both germline DNA, using a clinic-based series of sporadic and familial melanomas from South Italy (including cases from the entire Sardinia island), and somatic DNA, using a subset of melanoma tissues and cell lines. Correlations between genetic alterations and melanoma susceptibility or pathogenesis was thus inferred.

## Methods

### Melanoma cases

Eight hundred and forty-six patients with histologically-proven diagnosis of cutaneous melanoma were included into the study. Among them, 301 originated from Sardinia and 545 from other southern Italian regions; no substantial difference was observed in patients' characteristics between the Sardinian and non-Sardinian series. To avoid any bias, patients were consecutively collected from January 2001 to December 2006; they were included regardless of age of onset, cancer family history, and disease characteristics. Moreover, 35 melanoma tissues (paraffin-embedded primary melanomas and frozen samples from synchronous or asynchronous lymph node metastases of the same patients) and 18 melanoma cell lines (cultured from primary and metastatic tumours - see below) were collected.

Clinical and pathological features such as histological classification (including Breslow thickness) and disease stage at diagnosis were confirmed by medical records, review of pathologic material, and/or pathology reports. Familial recurrence of melanoma was ascertained by using a questionnaire to interview probands about their first-, second-, and third-degree relatives. Melanoma families were identified according to standardized criteria [23]: *a*) at least three affected members, or *b*) two affected members and presence of at least one of the following subcriteria: *i*) at least one affected member younger than 50 years at onset, or *ii*) a case of pancreatic cancer in a first- or second-degree relative, or *iii*) one affected member with multiple primary melanomas. Patients were informed about aims and limits of the study and blood samples were obtained with their written consent.

The study was reviewed and approved by the ethical review boards of both Azienda USL1 and University of Sassari.

### Mutation screening of candidate genes

For mutation analysis, genomic DNA was isolated from peripheral blood samples or tumour tissues or melanoma cell lines, using standard methods. At germline DNA level, the full coding sequences and splice junctions of *p16*^*CDKN*2*A *^(exons 1α, 2, and 3) and *BRCA2 *(exons 1-26) genes were screened for mutations using denaturing high-performance liquid chromatography (DHPLC) on a Wave^® ^nucleic acid fragment analysis system (Transgenomic, Santa Clara, CA). As previously reported by our group[28], abnormal PCR products identified by DHPLC analysis were directly sequenced using an automated fluorescence- cycle sequencer (ABIPRISM 3130, Applied Biosystems, Foster City, CA). About half of the present cohort (437 patients) has been already tested for *p16*^*CDKN*2*A *^germline mutations in our previous study [11]. Subsets of Sardinian patients originating from all geographical areas within the island were screened for mutations in the *MC1R *gene as above. At somatic DNA level, the full coding sequences and splice junctions of *p16*^*CDKN*2*A*^, *NRAS *(exons 2-3), and *BRAF *(exons 2-17) genes were screened for mutations as above. Primer sets and protocols for polymerase chain reaction (PCR) assays were as previously described [11,20,28,29]. For melanoma cell lines, we also performed mutation analysis of the *PTEN *gene (exons 1-9); primer sequences were as reported in Genome DataBase (GDB at http://www.ncbi.nlm.nih.gov/genome/guide/human).

To evaluate the prevalence of each gene variant in a control population, unrelated healthy individuals, originating from the same geographical areas and with no recurrence of cancer in family, were used as controls and screened for sequence variations.

### Immunochemical analysis

Immunocytochemistry was performed on cultured melanoma cells, using standard procedures. Melanoma cell lines were kindly provided by Dr. Stefania D'Atri at the Institute *Dermopatico dell'Iacolata *in Rome. They were established as primary short term cell cultures starting from tumour samples of donors patients with documented diagnosis of melanoma, after obtaining their informed consent Immunocytochemical analysis was performed using primary monoclonal antibodies against p16^CDKN2A ^(JC-2 Lab Vision-Neo-Markers, Fremont, CA) and pERK_1-2 _(Santa Cruz Biotechnology, Santa Cruz, CA) proteins.

Immunocytochemical staining was evaluated semi-quantitatively, using antibody negative and positive controls. Intensity and distribution of immunostaining was used to classify cells as positive (strong [+++] to moderate [++] staining, homogeneously distributed or presented by large majority of tumour cells) or negative (absent [-] or weak staining [+]) for gene expression. Scoring was performed by at least two investigators (in very few borderline cases, classification of immunostaining required additional investigators and was based on the consistency of the majority of them).

### Statistical analysis

Statistical correlation between *MC1R *germline mutations and disease was performed by chi-square test. Odds ratios of carrying gene mutations were estimated by the logistic regression model and are reported with 95% confidence interval (95% CI). Features for the relative risk calculation were analyzed as dichotomous variables (presence *versus *absence). The exact coefficient for sample proportion analysis was performed to determine all significant parameters (below 0.05 level). All analyses were performed using the statistical package SPSS/7.5 per Windows.

## Results

### Predisposing mutations in melanoma

Genomic DNA from 301 melanoma patients with ascertained Sardinian origin and 545 melanoma cases originating from non-Sardinian southern Italian regions was screened for germline mutations of *p16*^*CDKN*2*A*^, the main melanoma susceptibility gene. PCR products corresponding to the coding exons and intron-exon junctions were analyzed for mutations as described in Methods. For *p16*^*CDKN*2*A *^gene, 7 germline mutations were detected in 16/545 (2.9%) non-Sardinian patients (Table 1). All mutations have been reported as disease-related variants in the Human Gene Mutation Database at http://www.hgmd.cf.ac.uk; the S12X and E26X mutations were classified as disease-causing variants due to their truncation effect on proteins. In particular, six (17.1%) *p16*^*CDKN*2*A *^mutation carriers were among 35 melanoma patients who had at least one additional affected members in the family; the remaining ten (2.0%) *p16*^*CDKN*2*A *^mutation carriers were among 510 melanoma patients with sporadic disease (Table 2). None of 301 Sardinian patients was found positive for germline mutations in such a gene (Table 2). All *p16*^*CDKN*2*A *^mutations were absent in normal genomic DNA from 103 unrelated healthy individuals (corresponding to 206 control chromosomes), originating from the same geographical areas.

**Table 1:** Germline mutations in p16^*CDKN*2*A *^and BRCA2 genes.

Gene	Nucleotide	Codon	Base Change	Amino Acid Change	Designation
*P16*^*CDKN*2*A*^	35	12	C to A	Ser to Stop	S12X
*P16*^*CDKN*2*A*^	71	24	G to C	Arg to Pro	A24P
*P16*^*CDKN*2*A*^	76	26	G to T	Glu to Stop	E26X
*P16*^*CDKN*2*A*^	106	36	G to A	Ala to Thr	A36T
*P16*^*CDKN*2*A*^	176	59	T to G	Val to Gly	V59G
*P16*^*CDKN*2*A*^	301	101	G to T	Gly to Trp	G101W
*P16*^*CDKN*2*A*^	326	109	C to T	Ala to Val	A109V
*BRCA2*	8503	2835	T to C	Ser to Pro	Ser2835Pro
*BRCA2*	8765	2845	delAG	Stop 2867	8765delAG

**Table 2:** Distribution of germline mutations in p16^*CDKN*2*A *^and BRCA2 genes according to patients' origin

*Analyzed gene* Patients' origin	No. of analyzed patients	Positive cases	
		No.	%
***CDKN2A***	***846***	***16***	***1.9***
**non-Sardinian**	**545**	**16**	**2.9**
*Familial*	*35*	*6*	*17.1*
*Sporadic*	*510*	*10*	*2.0*
**Sardinian**	**301**	**0**	
*Familial*	*10*	*0*	
*Sporadic*	*291*	*0*	
***BRCA2***	***455***	***4***	***0.9***
**non-Sardinian**	**154**	**0**	
**Sardinian**	**301**	**4**	**1.3**
North Sardinia	91	4	4.4
*Familial*	*4*	*1*	*25.0*
*Sporadic*	*87*	*3*	*3.4*
Middle-South Sardinia	210	0	
*Familial*	*6*	*0*	
*Sporadic*	*204*	*0*	

The *p16*^*CDKN*2*A *^remained a melanoma susceptibility gene for non-Sardinian patients only; its involvement into the disease was absent among Sardinian patients. Therefore, we investigated whether additional candidate genes, such as *BRCA2 *and *MC1R*, might play a role in melanoma susceptibility within such an isolated population. Again, mutation screening for all coding regions and splice boundaries of *BRCA2 *and *MC1R *genes was performed as above.

Four (1.3%) out of 301 Sardinian patients were found to carry germline mutations in coding regions of the *BRCA2 *gene (three cases with BRCA2-8765delAG and the remaining one with BRCA2-Ser2835Pro); conversely, no *BRCA2 *mutation was observed in a subset of 154 non-Sardinian patients (to avoid any bias, mutation analysis for *BRCA2 *gene was performed on first 154 consecutively-collected cases from South Italy) (Table 2). Interestingly, all *BRCA2 *mutations of our series were detected in the subset of patients originating from North Sardinia (4/91; 4.4%); one *BRCA2*-positive case was among 4 patients with familial recurrence of melanoma, whereas the remaining three (3.4%) *BRCA2*-positive cases were among 87 patients with sporadic melanoma (Table 2). Both *BRCA2 *mutations were classified as disease-causing variants due to their predicted effect on proteins; they have been previously reported into the Breast Cancer Information Core (BIC) database at http://research.nhgri.nih.gov/bic/. Again, all *BRCA2 *mutations were not detected in normal genomic DNA from 103 unrelated Sardinian healthy individuals (corresponding to 206 control chromosomes). Cases with either familial or sporadic melanoma originating from other areas of the Sardinia island presented no germline mutation in *BRCA2 *gene (Table 2).

The entire coding region of the *MC1R *gene was then screened for germline sequence variations in 269 Sardinian melanoma patients (32 cases of our series were excluded because of DNA degradation or low amount of available genomic DNA) and 102 control subjects who were chosen as representative of the individuals living in the same geographical area and comparable for sex, age, general phenotype, and phototype to melanoma patients. Overall, 20 different *MC1R *variants were found; they were classified according to the effect of the gene sequence variations on the protein function [partial (r) or complete (R) loss of the receptor's signalling ability, as previously reported [20] (Table 3). Two *MC1R *germline variants classified as "R", Arg151Cys [odds ratio (OR), 6.4; 95% confidence interval (95% CI), 2.1-15.9] and Asp294 His (OR, 1.8; 95% CI, 1.1-5.3), were significantly associated with melanoma in our series (Table 3). Considering the individuals who were either homozygous or heterozygous for the "R" variant of the *MC1R *gene, the occurrence of such a "R" allele into the genotype was significantly associated with melanoma (p = 0.043; OR, 2.3; 95% CI, 1.2-7.8) (Table 4). No phenotypic parameter in our series of melanoma patients [sex, age of onset, primary tumour location, stage of disease, family history of melanoma, or geographical origin (North *vs*. Middle-South Sardinia)] was statistically correlated with the presence of the "R" genotype in *MC1R *gene (data not shown).

**Table 3:** Distribution of MC1R variants among Sardinian melanomas and controls

MC1R variants	Landi's classification	Positive cases (N = 269)	%	Positive controls (N = 102)	%	P
Val60leu (V60L)	R	88	32.7	32	31.4	n.s.
Ser83Pro (S83P)	R	3	1.1	1	1.0	n.s.
Asp84Glu (D84E)	R	1	0.4	0	0	n.s.
Val92Met (V92M)	R	16	5.9	5	4.9	n.s.
Thr95Met (T95M)	R	3	1.1	1	1.0	n.s.
Gly104Ser (G104S)	R	2	0.7	1	1.0	n.s.
Arg142Cys (R142C)	R	1	0.4	0	0	n.s.
Arg142His (R142H)	R	9	3.3	3	2.9	n.s.
**Arg151Cys (R151C)**	**R**	**19**	**7.1**	**1**	**1.0**	** > 0.01**
Tyr152Term (Y152Term)	R	1	0.4	0	0	n.s.
Ile155Thr (I155T)	R	4	1.5	2	2.0	n.s.
**Arg160Trp (R160W)**	**R**	**13**	**4.8**	**3**	**2.9**	**0.136**
Arg163Gln (R163Q)	R	7	2.6	2	2.0	n.s.
Ser172Ile (S172I)	R	1	0.4	0	0	n.s.
Arg213Trp (R213W)	R	5	1.9	1	1.0	n.s.
Ile221Thr (I 221 T)	R	1	0.4	0	0	n.s.
Pro256Ser (P256S)	R	2	0.7	0	0	n.s.
**Asp294His (D294H)**	**R**	**31**	**11.5**	**5**	**4.9**	**0.048**
Thr308Met (T308M)	R	2	0.7	0	0	n.s.
InsA724	R	2	0.7	0	0	n.s.

*MC1R *variants with complete loss of function are defined as "R" (in bold), whereas variants with partial loss of function are defined as "r". P values (chi-squared test; two tailed; 95% confidence interval) were given for *MC1R*-"R" variants only. n.s., not significant

**Table 4:** Frequency of R-containing genotypes in MC1R gene among Sardinian melanomas and controls

	Positive cases		
Subgroups (No. of subjects)	No.	%	*P*
***Patients ***(269)			
*R/R *or *R/r *or *R/wt *genotypes	**63**	23.4	*0.041*
***Controls ***(102)			
*R/R *or *R/r *or *R/wt *genotypes	**9**	8.8	

### Pathogenetic alterations in melanoma

A high prevalence of somatic mutations was detected in a subset of paired primary and secondary (lymph node metastasis) melanomas from 35 patients of the series (Table 5). Confirming previously reported data, *BRAF *and *NRAS *mutations were mutually exclusive in our patients' collection; overall, *BRAF *or *NRAS *mutations were detected in 23/34 (68%) primary tumours and 24/35 (69%) lymph node metastases from the same melanoma patients (Table 5). With the exception of one *BRAF *mutation (L597R) occurred in metastatic sample only, no difference in rates and types of mutations in *BRAF *and *NRAS *genes was observed between primary and secondary tumour tissues from same patients (Table 5). The rate of mutations in *p16*^*CDKN*2*A *^gene was found to instead increase from primary to metastatic melanomas of the same patients [5/33 (15%) to 8/35 (23%), respectively] (Table 5); in this sense, an even higher prevalence of *p16*^*CDKN*2*A *^alterations [8 (44%) gene mutations or hemi-homozygous exon deletions; 11 (61%) gene down-regulations] was observed in our series of 18 melanoma cell lines (Table 6). Conversely, a quite similar rate of *BRAF-NRAS *mutations (11/18; 61%) was detected in melanoma cell lines when compared to the uncultured melanomas (Table 6). Again, all detected mutations have been previously reported in the Human Gene Mutation Database at http://archive.uwcm.ac.uk.

**Table 5:** Somatic mutations in BRAF, NRAS, and p16^*CDKN*2*A *^genes among in vivo melanoma tissues

*Case*	*Sex*	*Age at diagnosis*	*Primary tumour*			*Lymph node metastasis*		
			*BRAF*	*NRAS*	*p16*^*CDKN*2*A*^	*BRAF*	*NRAS*	*p16*^*CDKN*2*A*^
MN01	M	43				L597R		
MN02	F	63		**Q61R**			Q61R	
MN03	M	51	**V600E**			V600E		
MN04	M	40	**V600E**			V600E		
MN06	M	49	**V600E**			V600E		
MN07	M	39	**V600E**			V600E		
MN08	F	65		**Q61R**			Q61R	
MN09	M	34		**Q61R**			Q61R	
MN10	M	67	**V600E**		**Arg24Pro**	V600E		Arg24Pro
MN11	M	84	**V600K**			V600K		
MN12	M	74						
MN14	M	41			**n.t.**			
MN15	M	63						Arg80ter
MN18	M	75						
MN19	M	30	**V600E**			V600E		
MN20	F	45	**V600E**			V600E		
MN21	M	35			**Ala36Thr**			Ala36Th
MN22	M	71		**Q61R**			Q61R	
MN25	F	35	**V600E**			V600E		
MN26	M	50		**Q61R**			Q61R	
MN27	F	48						
MN28	F	58		**Q61K**			Q61K	
MN29	M	51	**V600E**			V600E		
MN31	M	40		**Q61R**			Q61R	
MN34	F	65		**Q61R**	**Ala109Val**		Q61R	Ala109Val
MN35	M	61						
MN36	M	64			**Trp110ter**			Trp110ter
MN41	F	69						
MN45	F	56	**n.t.**	**n.t.**	**n.t.**			Arg80ter
MN05	M	51	**V600E**			V600E		IVS1+1G>A
MN55	F	34						
MN56	F	36	**V600E**			V600E		
MN59	F	53		**Q61K**			Q61K	
MN68	M	45		**Q61R**			Q61R	
MN75	F	56		**Q61R**	**Arg24Pro**		Q61R	Arg24Pro

Primary melanomas are in bold. n.t., not tested

**Table 6:** Genetic and functional alterations in candidate genes among in vitro melanoma cell lines

Cell line *derived from*	ERK1	ERK2	pERK	p16	BRAF	NRAS	p16^CDKN2A^
*Primary melanoma*							
**GR-Mel**	+++	++	+	++			
**LCP-Mel**	+++	+++	**++**	**+**	**V600R**		**Del exon 2**
**MNG**	+++	+++	**++/+++**	**+**			
**PNP-Mel**	+++	+++	**++**	**+**	**V600E**		**G101W**
**ST-Mel**	++/+++	+++	**+++**	++	**G466E**		
*Metastatic lymph node*							
**CN-Mel**	+++	+++	**++**	**+**		**Q61R**	
**CR-Mel**	+++	+++	**++**	**+**		**Q61K**	**Del exon 2**
**GL-Mel**	+++	+++	**++**	**+**			**Del exon 2**
**LCM-Mel**	+++	++	**++**	**-**	**V600R**		**Del exons 1-2**
**MAR**	+++	+/++	+	**+**			
**SK-Mel-28**	+++	++	**++**	+++	**V600E**		
**13443-Mel**	+++	++	+	**+++**			
*Cutaneous metastasis*							
**LB-24-Mel**	+++	+++	**+++**	++			
**M14**	+++	+++	**++**	**+**	**V600E**		**455insCdel26 IVS1+2T>C**
**PR-Mel**	+++	+++	+	++	**V600R**		
**SN-Mel**	++	++/+++	+	++	**V600E**		
**WM-266-4**	+++	++	**++/+++**	**-**	**V600D**		**Del exon 1**
**397-Mel**	++	++	**++**	**+**			**Del exon 2**

### No *PTEN *mutation was found in melanoma cell lines

**Figure 1:**
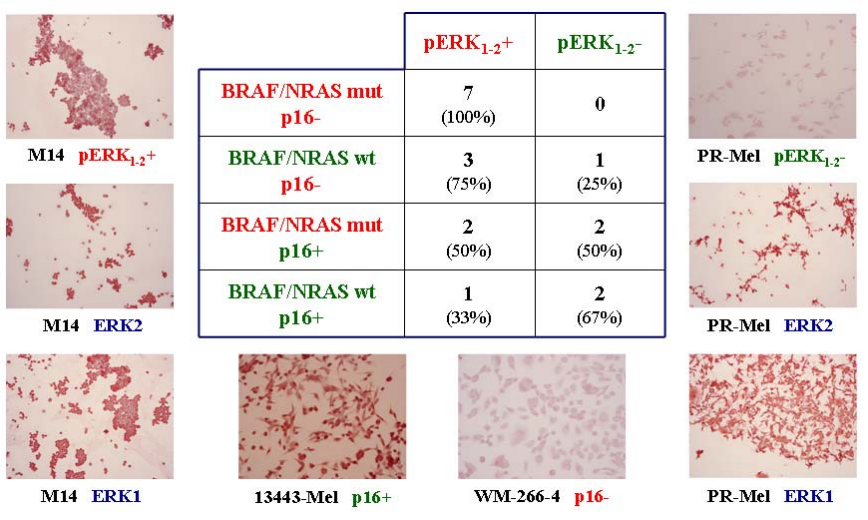
**Classification of melanoma cell lines according to alterations in *NRAS*/*BRAF *genes and p16^CDKN2A^/pERK_1-2 _protein expression**. Data regarding occurrence of mutations in *BRAF*/*NRAS *genes (mut), down-regulation of p16^CDKN2A ^protein (p16-), and over-expression of phosphorilated ERK_1-2 _protein (pERK_1-2_+) are indicated. Exemplificative immunochemical results are shown.

**Figure 2:**
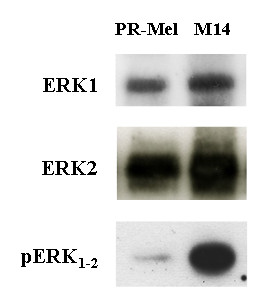
**Western blot analysis of M14 and PR-Mel melanoma cell lines**. Protein lysates from M14 and PR-Mel cells were resolved by SDS-PAGE gel electrophoresis and transferred to a nylon membrane; the proteins on the membrane were then subjected to immunoblot analysis with antibodies against ERK1-2/pERK_1-2 _proteins.

Considering the immunocytochemical results, all melanoma cell lines (7/7; 100%) with concurrent mutation of *BRAF*-*NRAS *and down-regulation of *p16*^*CDKN*2*A *^presented high expression levels of the activated pERK_1-2 _protein (Figure 1). The remaining subsets of melanoma cell lines with alterations of one or none of such genes presented lower rates of ERK1-2 phosphorilation [5/8 (62%) and 1/3 (33%), respectively] (Figure 1), suggesting that coexistence of multiple molecular events may be required for activating ERK in melanoma. In Figure 2, the expression of ERK1, ERK2, and pERK_1-2 _proteins was validated by Western blot hybridization in M14 and PR-Mel melanoma cell lines.

## Discussion

Melanocytic transformation is thought to occur by sequential accumulation of alterations in several genes and metabolic pathways [8,30,31]. In our study, we tried to better define the role of the genes mainly involved in melanoma susceptibility (through the assessment of the prevalence of predisposing germline mutations) and pathogenesis (by investigating somatic gene down- or up-regulations in tumour tissues and cancer cell lines).

### Melanoma susceptibility

Assessment of the prevalence of predisposing germline mutations in candidate genes represents an important step toward prevention and early detection of cancers. In this sense, the mutation analysis of *p16*^*CDKN*2*A*^, which is the main gene associated with melanoma susceptibility [8,30], should be considered as a crucial step. Due to environmental and genetic factors, prevalence of *p16*^*CDKN*2*A *^mutations has been demonstrated to deeply vary among different populations [10]. A real estimation of the proportion of positive and negative tests that might be expected in a referral risk evaluation clinic is therefore fundamental to provide clinical recommendations for *p16*^*CDKN*2*A *^genetic testing.

In our analysis, *p16*^*CDKN*2*A *^germline mutations were detected in about 3% (16; 2.9%) out of 545 melanoma patients from southern Italian regions not including Sardinia; in this island, none of the 301 analyzed patient was found to carry a *p16*^*CDKN*2*A *^mutation. When Sardinian patients were screened for germline mutations in *BRCA2 *gene, which seems to play a major role in predisposition to different types of cancer including melanoma (although data are still insufficient, annual skin and eye examinations for early diagnosis of melanoma have been proposed for genetic counselling of *BRCA2 *carriers) [11-14], we exclusively observed a mutated *BRCA2 *gene in patients originating from North Sardinia (4/91; 4.4%); no *BRCA2 *germline mutation was observed in 210 patients originating from the remaining areas of the island or in a subset of 154 non-Sardinian melanoma cases (see Table 2). Moreover, we obtained evidence that no sequence variation was present in *p15*^*CDKN*2*B *^and *CDK4*, the remaining two high penetrance melanoma susceptibility genes, among familial melanoma cases from our southern Italian population ([32] and unpublished data). Therefore, the central-southern part of the Sardinia island should be considered as a geographical area in which melanoma patients do not carry mutations in any known major susceptibility gene. Since the incidence rates of melanoma are quite similar in both Sardinian, genetically homogeneous, and non-Sardinian, genetically heterogeneous, populations from South Italy (roughly, 4 new cases per 100.000 inhabitants per year [5]), our findings seem to further confirm that genetic factors predisposing to melanoma are geographically heterogeneous and strictly depending on patients' origin. On this regard, it is worth to underline that Sardinia island has a relatively small and isolated population with a high rate of inbreeding; in comparison to the genetically-heterogeneous Italian population, a higher prevalence of mutations with founder effect has been reported (even for cancer diseases) [33,34]. Nevertheless, one could speculate that prevalence of mutations in melanoma susceptibility genes needs to be investigated in every different geographical area.

Considering the familial recurrence of melanoma, 48/846 (5.7%) patients from our series presented at least one additional affected member in the family. Overall, seven (14.6%) melanoma families had a detectable mutation in *p16*^*CDKN*2*A *^or *BRCA2 *gene. Also considering some lack of sensitivity of the mutation analysis approach, the prevalence of such germline mutations among patients with familial melanoma from South Italy remains low. However, vast majority of the families included into the present study contains two melanoma patients only; therefore, more stringent selection criteria or subcriteria should be used in order to reduce chances that the identified melanoma families may simply represent a cluster of sporadic cases. As a confirmation of this hypothesis, a consistent increase in prevalence rate of *p16*^*CDKN*2*A *^mutations was observed among families with three affected members (3/7; 42.9%). Although large genomic deletions may escape detection by direct sequencing, undetected mutations are unlikely to explain the high number of families without mutations. Therefore, these data further support the hypothesis that additional low-penetrance melanoma susceptibility genes remain to be identified.

Finally, our study confirmed the association of some *MC1R *gene variants with the occurrence of cutaneous melanoma as previously reported [15,35,36]. Despite the *MC1R *sequence screening was carried out among Sardinian patients only (due to the availability of control subjects representative of the individuals living in the island and comparable for phenotypic features to melanoma patients), the variants classified as "R" and, particularly, the Arg151Cys and Asp294His alleles appeared to be associated with melanoma. Considering the functional outcome, both Arg151Cys and Asp294His variants have been demonstrated to affect the normal signalling of the melanocortin-1 receptor by reducing the ability to elevate the intracellular levels of cAMP [16]. Further and more specific studies (based on better classification of the different skin phototypes and/or a more detailed evaluation of the general phenotypic characteristics) are required in order to define the role of such *MC1R *gene variants into the susceptibility to melanoma.

### Melanoma pathogenesis

We here examined the relationship between alterations in *NRAS*, *BRAF*, and *p16*^*CDKN*2*A *^genes in both *in vivo *melanoma tissues (N = 35) from southern Italian patients and *in vitro *melanoma cell lines (N = 18). We found that mutually exclusive mutations of *NRAS *and *BRAF *genes occur at quite same rate in cultured and uncultured melanomas (either primary or metastatic lesions), confirming that they represent an early event within the cascade of alterations involved into the melanomagenesis. However, distribution of mutations in each gene (*NRAS *or *BRAF*) deeply varied into the analyzed somatic samples from our series (see Tables 5 and 6), suggesting that pathogenetic alterations may indifferently affect kinases acting either up- or downstream within such a signalling cascade. Moreover, no concurrent mutation of the *PTEN *gene was observed in melanoma cell lines; this is clearly in contrast with previous data from other series, which reported that such a gene is mutated at a rate of about 30% among *in vitro *melanomas [37]. Conversely, *p16*^*CDKN*2*A *^gene mutations and/or rearrangements (mostly, represented by exon deletions) were observed at increased rates moving from primary to metastatic melanomas and melanoma cell lines (see Tables 5 and 6). Down-regulation or inactivation of the *p16*^*CDKN*2*A *^gene (in our series, about two thirds of melanoma cell lines presented a reduced or absent expression of the *p16*^*CDKN*2*A *^protein) has been demonstrated to affect the control of cell growth, which may induce cell proliferation and increase aggressiveness of transformed melanocytic cells (melanoma cells tend to inactivate both alleles of such a tumour suppressor gene) [38].

Presence of *BRAF *mutations in benign and dysplastic nevi [39] supports the hypothesis that activation of the *NRAS*-*BRAF*-*ERK *pathway is not sufficient to induce the malignant process and fully transform proliferating melanocytes, but requires additional, cooperating de-regulative events. In our series, the increased activity of ERK1/2 proteins was mainly a consequence of a combination of mutations in upstream *NRAS*/*BRAF *components of the pathway and silencing of the *p16*^*CDKN*2*A *^gene (although additional - and yet unidentified - functional alterations may participate in inducing such a ERK activation) (see Figure 1). Therefore, our data provide an additional confirmation that multiple molecular events are being accumulated during melanomagenesis.

Identification of the predominant germline mutations in candidate susceptibility genes within a particular geographical area has particular relevance to achieve a prediction of the melanoma risk as well as to address patients and their families to clinical screening. On this regard, a dramatic improvement toward an earlier diagnosis of melanoma could be represented by the selection of specific high-risk groups to be appropriately targeted (since routine screening for detection of thinner melanoma can not be indiscriminately proposed). Familial melanoma patients are reported to present with thinner melanomas [8]. As also indicated above, different classifications of familial melanoma have been used by several authors, based on number (two cases with additional and various subcriteria or at least three cases) and type (involvement of first- and/or second-degree relatives) of affected family members. Regardless the occurrence of a weak or strong family history, relatives of melanoma patients carrying germline mutations in susceptibility genes could represent a high risk group which might undergo a surveillance program for identification of thinner melanoma. Our experience seems to support this hypothesis. Considering the non-Sardinian melanoma patients (whose series was the only one in which we detected *p16*^*CDKN*2*A *^germline mutations), first- and second-degree relatives of *p16*^*CDKN*2*A *^mutation carriers were informed to belong to a putative high risk group (through specific educational sessions, after obtaining an informed consent) and addressed to a short-term (6 months) surveillance program using epiluminescence microscopy. After a median follow-up of 78 months (range, 37-109), six new melanomas with median Breslow thickness of 0.35 mm (range, 0.25-0.68) were observed among such relatives of *p16*^*CDKN*2*A *^mutation-positive patients (it is important to underline that patients with very thin melanomas - Breslow thickness ≤ 0.40 mm - present a 10-year survival rate which is estimated to be more than 98% [40,41]). Although the number of events for more mature and definitive results is really low, we could speculate that the identification of a melanoma patient carrying a *p16*^*CDKN*2*A *^germline mutation should induce clinicians to educate his family members to have a great care of all skin lesions and pay high attention to noticing any nevi' modification as well as to address them to a routine screening for detection of thinner melanoma.

## Conclusion

Although most genetic and molecular alterations have been identified, characterization of all interactions between key effectors in MAPK, CDKN2A, and additional (i.e. PTEN-AKT) pathways will represent the aims of future research efforts, in order to further clarify the sequence of events inducing transformation of melanocytes and progression of melanoma.

## Competing interests

The authors declare that they have no competing interests.

## Authors' contributions

MC performed all mutation analyses. AM participated to patients' collection. AC participated to the collection of somatic samples. MB participated to analysis and interpretation of data. CC participated to patients' collection. PAA participated to patients' collection. EP participated to characterization of melanoma cell lines. IS participated to patients' collection. SC participated to patients' collection. MS performed immunochemical analyses. GrP participated to mutation analysis and cell biology. GiP conceived of the study and drafted the manuscript.

All authors read and approved the final manuscript.

## Pre-publication history

The pre-publication history for this paper can be accessed here:

http://www.biomedcentral.com/1471-2407/9/352/prepub
